# Development and application of a questionnaire to assess patient beliefs in rheumatoid arthritis and axial spondyloarthritis

**DOI:** 10.1007/s10067-018-4172-5

**Published:** 2018-06-12

**Authors:** Laure Gossec, Francis Berenbaum, Pierre Chauvin, Christophe Hudry, Gabrielle Cukierman, Thibault de Chalus, Caroline Dreuillet, Vincent Saulot, Sabine Tong, Françoise Russo-Marie, Jean-Michel Joubert, Alain Saraux

**Affiliations:** 1 Sorbonne Université, Institut Pierre Louis d’Epidémiologie et de Santé Publique (INSERM UMRS 1136), GRC-UPMC 08 (EEMOIS), Paris, France; 2 Service de rhumatologie, Hôpital Pitié Salpêtrière, 47–83 Boulevard de l’Hôpital, 75013 Paris, France; 3 Sorbonne Université, INSERM, Paris, France; 4 Department of Rheumatology, Hôpital Saint-Antoine, AP-HP, Paris, France; 5 Sorbonne Université, INSERM, Institut Pierre Louis d’Epidémiologie et de Santé Publique (UMRS 1136), Department of Social Epidemiology, Sorbonne Universités, Paris, France; 6 Department of Rheumatology, Hôpital Cochin, AP-HP, Paris, France; 7 UCB Pharma, Colombes, France; 8 Arthritis Fondation Courtin, Neuilly-Sur-Seine, France; 9 AXONAL-BIOSTATEM, Nanterre, France; 10 Department of Rheumatology, CHU de la Cavale-Blanche, Brest, France; 11 INSERM, LabEx IGO, UMR1227, Lymphocytes B et Autoimmunité, Université de Brest, Brest, France

**Keywords:** Behavior, Outcome measures, Patient attitude to health, Rheumatoid arthritis, Spondyloarthritis

## Abstract

Misinterpretation of patient beliefs may complicate shared decision-making in rheumatoid arthritis (RA) or axial spondyloarthritis (axSpA). The objective of this study was to develop a questionnaire to assess patients’ beliefs about their disease and its treatment, and to identify patient characteristics associated with these beliefs. All beliefs reported by > 5% of 50 patients in a previous study were reformulated with a partnering patient organization into statements with which participants could rate their agreement on a scale of 0–10 (totally disagree to totally agree). The resulting Questionnaire for Arthritis Dialogue (QuAD) was made available to patients with RA or axSpA. A score ≥ 7 was considered a strongly held belief. Associations between patient characteristics and individual lifestyle beliefs were assessed using multiple logistic regression. The 21-item QuAD was completed by 672 patients (432 RA, 240 axSpA; mean [±SD] age 54.2 [± 14.2]; 63.7% female). The most widely held beliefs were related to uncertainty about progression (*n* = 354, 54.0%), heredity (*n* = 309, 47.8%), and flare triggers (*n* = 283, 42.7%). The unwarranted belief that physical activity is deleterious to disease activity was associated with markers of psychological distress and lower educational levels. The beliefs of patients with RA or axSpA about their disease are wide-ranging. Since these may be unwarranted and may lead to inappropriate behaviors, physicians should discuss these beliefs with their patients. The QuAD may facilitate this dialogue, and may also be useful in population studies to standardize the assessment and evolution of beliefs over time. People with long-term inflammatory conditions such as rheumatoid arthritis (RA; inflammation of the joints) and axial spondyloarthritis (axSpA; inflammation of the spine) may hold a number of beliefs about their disease, including some that are not supported by current scientific evidence (e.g., “I think that my disease was triggered by a vaccination”). Some beliefs, especially those relating to the role of lifestyle factors (such as exercise, diet, smoking, and drinking alcohol), may encourage people living with severe diseases to change their behavior in a way that has an effect on their disease. Within this project, we developed a questionnaire to identify the most common beliefs held by people living with RA or axSpA, which is called the “Questionnaire for Arthritis Dialogue (QuAD).” We also examined whether certain characteristics (or traits) of people living with RA or axSpA are linked to beliefs not currently supported by scientific evidence. A total of 672 people living with RA or axSpA in France were asked to complete the questionnaire (QuAD). The questionnaire included 21 opinion statements that they scored from 0 (totally disagree) to 10 (totally agree). A score of more than 7 was interpreted to mean that the person significantly agreed with the opinion. Based on the responses to specific opinion statements in the questionnaire, we were able to identify possible links between beliefs that are not supported by scientific evidence (e.g., “I think that flare-ups of my disease are triggered by physical effort”), and characteristics of people living with severe diseases. Our findings suggested that beliefs about lifestyle and inflammatory diseases varied from person to person, were sometimes inconsistent (the most widely held beliefs were sometimes contradictory), and were often not supported by scientific evidence. The belief that physical activity had negative effects on the disease was linked to poor education and psychological issues (such as anxiety and helplessness). People living with axSpA were more likely to believe their disease was a result of their genetic make-up, whereas those with RA more often believed their disease was caused by emotional issues. People living with axSpA were also more likely to believe that physical activity could be beneficial for their disease, and less likely to believe that their disease was caused by smoking. Our results suggest that doctors need to discuss with their patients how they might believe lifestyle is associated with their disease. This will help to dispel any unnecessary concerns, and to encourage their patients to take up healthy lifestyles and habits that are beneficial for their disease management. It may also be beneficial for health care providers to discuss the beliefs identified in this study during educational programs about inflammatory diseases, for the benefit of people living with RA or axSpA.

**Electronic supplementary material:**

The online version of this article (10.1007/s10067-018-4172-5) contains supplementary material, which is available to authorized users.

## Introduction

Chronic inflammatory rheumatic diseases (CIRDs), of which rheumatoid arthritis (RA) and axial spondyloarthritis (axSpA) are the most frequent, are progressive diseases that evolve with an unpredictable and fluctuating course over the patient’s lifetime. The chronic nature of these diseases, the heterogeneity of physical manifestations between patients, and the difficulty in foreseeing disease flares and long-term progression create uncertainty and stress for the patient. In addition, they make it difficult for patients to develop a valid internal representation of their disease [1]. This may in turn lead to misplaced disease perceptions and treatment expectations [2, 3], and the development of inappropriate behaviors for managing disease manifestations and coping with their consequences [4, 5]. For example, patients who believe that disease flares are triggered by physical activity may actively pursue a sedentary lifestyle, with detrimental consequences for their CIRD and their general health [6]. The erroneous assumption that diet has an impact on disease may also lead the patient to make poor nutritional choices [7]. In addition, expectations about the benefits and risks of treatment with disease-modifying anti-rheumatic drugs (DMARDs) may influence treatment adherence [8] or perceptions of tolerability [9].

Therefore, it is important for physicians to understand their patients’ beliefs about their disease, and to initiate a dialogue with the patient about unwarranted beliefs, with the goal of modifying behavior and thereby improving overall health. However, very little research has been published relating to the beliefs and apprehensions of patients with CIRDs [10].

In order to gain more information about disease perceptions in patients with CIRDs, a research program was initiated, with the aim of developing a questionnaire to evaluate these beliefs. The specific objectives of this study were to develop a questionnaire to assess beliefs in patients with RA or axSpA regarding their disease and its treatment, and to identify patient characteristics associated with these beliefs.

## Materials and methods

### Development of the questionnaire for arthritis dialogue

A previous qualitative study of disease perception in patients with RA or axSpA was performed in 50 patients (25 with RA and 25 with axSpA) [11]. Based on data from this study [11], all items reported by > 5% of patients were rephrased as assertions, with help from a partnering patient organization. This questionnaire covered the most widely held perceptions about disease and treatment. Other questions related to patient fears and beliefs are reported elsewhere [12]. Each item was scored on a 10-point numerical rating scale (NRS) ranging from 0 (totally disagree) to 10 (totally agree).

The questions were tested in a sample of 10 patients for linguistic validation and cognitive debriefing. The original French questionnaire was translated into English through two independent forward translations (French to English) followed by two independent back translations (English to French), with reconciliation of the translated texts [13]. The questionnaire took around 25 min to complete.

### Application of the questionnaire for arthritis dialogue to a wide sample

This cross-sectional, prospective study included patients with RA or axSpA in everyday practice in France, and was implemented by hospital and community rheumatologists between July 2014 and October 2015. The study was performed in accordance with Good Epidemiological Practice [14] and relevant French guidelines for patient surveys. Verbal informed consent was obtained from all participating patients. The study protocol was considered by the Ethics Committee of the St Antoine Hospital, Paris, to be both ethical and outside the scope of French legislation restricting biomedical research (session of 7th October 2014). The study was also declared to the National Advisory Committee on Medical Research Information (CCTIRS) and the French national data protection agency (CNIL).

All rheumatologists currently practicing in France were invited to participate in the study though post and email. Each participating rheumatologist was expected to invite all consecutive patients with RA or axSpA during routine outpatient visits who fulfilled the eligibility criteria (up to 20 patients per investigator). Adult patients (aged > 18 years) with a diagnosis of RA according to the American College of Rheumatology/European League Against Rheumatism (ACR/EULAR) classification criteria [15], or of axSpA according to the Assessment in Spondyloarthritis International Society (ASAS) classification criteria [16], were eligible and were enrolled if they agreed to participate. Patients who were unable to complete a questionnaire in French were excluded.

### Data collection

Patients were asked to complete both the beliefs questionnaire and other questions relating to fears (44 items in total) [12], the Hospital Anxiety and Depression Scale (HADS) [17], the Arthritis Helplessness Index (AHI) [18], the Patient Global Assessment (PGA) of overall disease activity (scored between 0 and 10), and for patients with axSpA, the Bath Ankylosing Spondylitis Disease Activity Index (BASDAI) [19]. All questionnaires were in French. Patients also provided information on sociodemographic indicators, health insurance coverage and disease duration.

In parallel, rheumatologists provided information on their own age, gender, type of practice (hospital, community, or mixed), and geographical region, in addition to information about the patient on current treatment, disease activity measured with the 28-item Disease Activity Score calculated with erythrocyte sedimentation rate (DAS28[ESR]) [20] for RA, and an overall assessment of disease activity scored from 0 to 10.

### Statistical analysis

Data were analyzed for all patients for whom both patient and physician questionnaires were available. For each item of the questionnaire, mean value ± standard deviation (SD) and the percentage of patients with scores ≥ 7 were determined both for the total population, and for the RA and axSpA subgroups. Comparisons were performed with the *χ*^2^ test (corrected for continuity) or Fisher’s exact test, as appropriate. A score of ≥ 7 was taken to indicate strong agreement with the opinion presented. This cut-off was chosen on an empirical basis; in the absence of any known disease characteristic with which these beliefs are correlated, we did not feel that it was realistic to attempt psychometric calibration of the VAS.

In order to identify variables associated with a score ≥ 7 for a given item, we performed a univariate regression analysis for all patient and physician variables documented in the study. Variables with an association at a probability threshold of 0.20 (*χ*^2^ test) were entered into a backwards stepwise multiple logistic regression analysis. A threshold of 0.05 was used for retention of variables in the model.

Multiple imputation methods (Markov chains using Monte Carlo simulations) were used for missing data when this concerned > 5% of all patients. When this proportion was ≤ 5%, missing data were replaced with the median value of the full study sample. All statistical analyses were performed using SAS® Version 9.2 (SAS Institute, Cary, NC, USA).

### Role of the funding source

UCB Pharma sponsored the study and the development of the manuscript and reviewed the text to ensure that from a UCB Pharma perspective, the data presented in the publication are scientifically, technically and medically supportable, that they do not contain any information that has the potential to damage the intellectual property of UCB Pharma, and that the publication complies with applicable laws, regulations, guidelines and good industry practice. The authors approved the final version to be published after critically revising the manuscript for important intellectual content.

## Results

### Characteristics of the questionnaire for arthritis dialogue

The Questionnaire for Arthritis Dialogue (QuAD) includes 44 items in total, 21 of which cover beliefs on the cause of disease, disease flares and treatments. The remaining 23 items concern fears that are described in detail elsewhere [21].

### Participants in the validation study

Of the 1618 rheumatologists in France who were contacted, 134 agreed to participate in the study (including 20 who were exclusively community-based, 51 exclusively hospital-based and 29 with a mixed practice), and 100 enrolled at least one patient.

A total of 796 patients were enrolled, of whom 672 (84.4%) were available for analysis (Table 1). The remaining patients were excluded, due to either missing physician (*n* = 98) or patient (*n* = 12) questionnaires, or because the diagnostic criteria for RA/axSpA were either not fulfilled (*n* = 5) or not documented (*n* = 10). The median number of patients enrolled by each center was six. Patients with RA were more frequently female, and older on average than those with axSpA. Both physician and patient global assessments were higher for patients with axSpA than for those with RA. In both groups, around three-quarters of patients were undergoing treatment with biological DMARDs.

**Table 1:** Patient characteristics

	RA [*n* = 432]	axSpA [*n* = 240]	Total [*N* = 672]
Age (years)	58.3 (13.1)	47.0 (13.2)	54.2 (14.2)
Gender (women, %)	276 (74.0%)	94 (45.2%)	370 (63.7%)
Professional activity			
In employment	162 (38.2%)	167 (70.5%)	329 (49.8%)
Retired	201 (47.4%)	30 (12.7%)	231 (34.9%)
Other	61 (14.4%)	40 (16.8%)	101 (15.3%)
Education level			
Primary	77 (18.0%)	11 (4.6%)	88 (13.3%)
Secondary	219 (51.3%)	134 (56.3%)	353 (53.1%)
Tertiary (post-high school)	131 (30.7%)	93 (39.1%)	224 (33.7%)
Disease duration (years)	13.1 (11.4)	13.8 (10.6)	13.4 (11.1)
Disease activity			
DAS28(ESR)	2.6 (1.2)	–	–
BASDAI	–	3.3 (2.2)	–
Physician global assessment (0–10)	2.75 (2.12)	3.44 (2.41)	3.00 (2.25)
Patient global assessment (0–10)	3.03 (2.45)	4.27 (2.61)	3.48 (2.58)
Treatments			
Corticosteroids alone	6 (1.8%)	–	6 (1.1%)
NSAIDs alone	–	36 (15.1%)	36 (6.4%)
Synthetic DMARDs ± corticosteroids/NSAIDs	61 (18.7%)	15 (6.3%)	76 (13.5%)
Biological DMARDs (alone or in combination)	252 (77.3%)	173 (72.7%)	425 (75.4%)
Other	2 (0.6%)	7 (2.9%)	9 (0.7%)

Data are presented as mean values (standard deviation) for continuous variables, and as frequency counts (%) for categorical variables. Data were missing for some patients for all variables

axSpA, axial spondyloarthritis; BASDAI, Bath Ankylosing Spondylitis Disease Activity Index; DAS28(ESR), 28-item disease activity score measured with erythrocyte sedimentation rate; DMARD, disease-modifying anti-rheumatic drug; NSAID, non-steroidal anti-inflammatory drug; RA, rheumatoid arthritis

### Beliefs of patients with rheumatoid arthritis and axial spondyloarthritis

The 21 relevant items in the QuAD **(**Table 2**)** included beliefs about psychological factors (2 items), genetic factors (2 items), physical activity (4 items), diet (4 items), and other lifestyle factors (3 items). The remaining items were categorized as miscellaneous beliefs (6 items).

**Table 2:** The QuAD questionnaire and mean scores for each item in patients with RA or axSpA

QuAD item		RA *n* = 432	axSpA *n* = 240	Total *N* = 672
	Psychological factors			
P1	I think that my disease was triggered by an emotional shock. (A difficult or stressful period in my life).	5.1 (3.9)	3.6 (3.8)	4.6 (3.9)
P2	I think that flare-ups of my disease are triggered by psychological factors (stress, upset, low morale, etc.).	4.6 (3.5)	4.7 (3.3)	4.7 (3.4)
	Genetic factors			
G1	I think that my disease has a genetic cause.	4.0 (3.6)	6.6 (3.5)	5.0 (3.8)
G2	I am afraid of passing my disease on to my children.	4.7 (4.1)	6.8 (3.7)	5.5 (4.1)
	Physical activity			
F1	I think that my disease was triggered by physical overload.	2.9 (3.3)	3.1 (3.3)	3.0 (3.3)
F2	I think that flare-ups of my disease are triggered by physical effort.	4.2 (3.5)	5.4 (3.2)	4.6 (3.4)
F3	I think that my flare-ups are triggered by bad posture or staying in the same position for too long.	3.2 (3.3)	5.7 (3.3)	4.1 (3.5)
F4	I think that doing sport or a physical activity reduces my flare-ups.	4.5 (3.3)	5.9 (3.0)	5.0 (3.3)
	Diet			
D1	I think that my disease may have been triggered by what I ate.	1.3 (2.3)	1.3 (2.3)	1.3 (2.3)
D2	I think that drinking alcohol (even moderately) triggered my disease.	0.7 (1.7)	0.5 (1.4)	0.7 (1.6)
D3	I think that eating certain foods triggers my flare-ups.	2.1 (2.9)	2.0 (2.7)	2.0 (2.8)
D4	I think that eating certain foods reduces my flare-ups.	2.2 (3.0)	1.9 (2.7)	2.1 (2.9)
	Other lifestyle factors			
O1	I think that my flare-ups are triggered by fatigue.	4.9 (3.4)	5.8 (3.1)	5.2 (3.3)
O2	I think that smoking (even moderately) or being exposed to passive smoking triggered my disease.	1.5 (2.5)	1.0 (1.8)	1.3 (2.3)
O3	I think that my disease was triggered by something in the environment, like pollution.	1.6 (2.5)	1.3 (2.4)	1.5 (2.5)
	Miscellaneous			
M1	I think that my flare-ups are triggered by a change in the weather.	4.3 (3.4)	5.1 (3.4)	4.6 (3.4)
M2	I think that my disease was triggered by a vaccination.	1.4 (2.7)	1.3 (2.7)	1.3 (2.7)
M3	I think that my disease was triggered by an infection.	1.7 (2.7)	1.7 (2.7)	1.7 (2.7)
M4	I think that some types of alternative medicine (osteopathy, acupuncture, sophrology, homeopathy, etc.) reduce my flare-ups.	3.5 (3.4)	3.8 (3.3)	3.6 (3.3)
M5	I think that all treatments have negative effects in the long term.	4.9 (3.5)	5.4 (3.1)	5.1 (3.4)
M6	I don’t know how my disease will progress (and that worries me).	5.9 (3.2)	7.0 (3.1)	6.3 (3.2)

Scores are presented as mean scores (standard deviation) on a scale from 0 to 10, where 10 indicates full agreement

axSpA, axial spondyloarthritis; QuAD, Questionnaire for Arthritis Dialogue; RA, rheumatoid arthritis

**Fig. 1:**
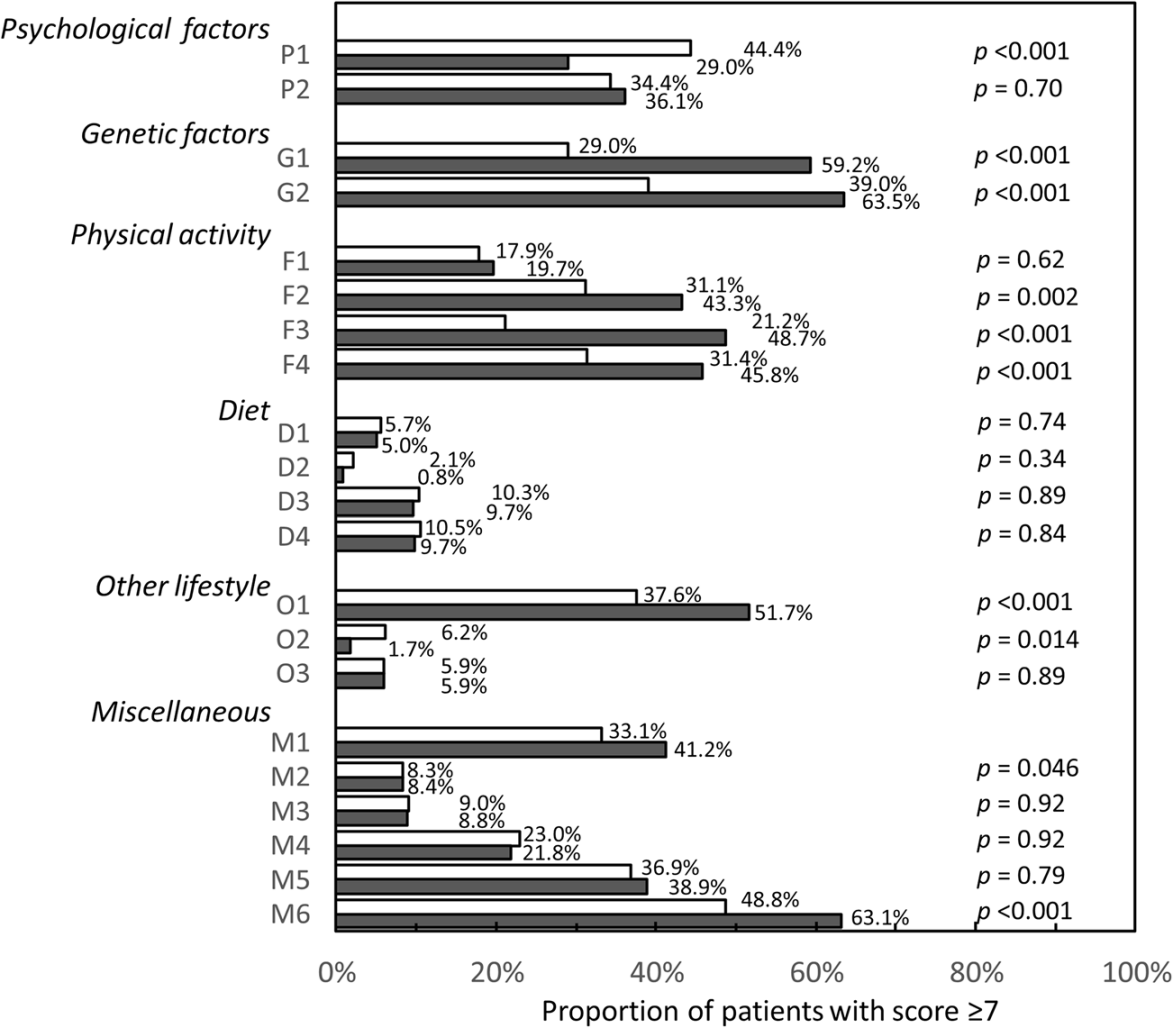
Proportion of patients with strongly held beliefs (QuAD score ≥ 7). The codes for the questionnaire items correspond to those listed in Table 2. □: patients with RA (*n* = 432); ■: patients with axSpA (*n* = 240). QuAD: Questionnaire for Arthritis Dialogue

Mean (±SD) scores for each item of the QuAD ranged from 0.7 (± 1.6) for “I think that drinking alcohol (even moderately) triggered my disease” to 6.3 (± 3.2) for “I don’t know how my disease will progress (and that worries me).” The proportion of patients rating each item of the QuAD with a score ≥ 7 is presented in Fig. 1. Overall, beliefs appeared to be more strongly held in patients with axSpA than in those with RA. The three most widely held beliefs were: “I don’t know how my disease will progress (and that worries me)” (*n* = 354, 54.0%), “I am afraid of passing my disease on to my children” (*n* = 309, 47.8%), and “I think that my flare-ups are triggered by fatigue” (*n* = 283, 42.7%).

### Characteristics of patients with specific lifestyle beliefs

A comprehensive listing of patient characteristics associated with individual lifestyle beliefs, identified from the univariate and multivariate analyses, is provided in Online Resource 1 (physical activity items), Online Resource 2 (food and diet items) and Online Resource 3 (other lifestyle items).

Findings from the multivariate analysis are presented in Table 3. The belief that “eating certain foods could reduce disease flares” had more acceptance in women than in men. The belief that “physical activity could reduce disease flares” had more acceptance in patients with higher education, while fewer patients in this subgroup believed that their disease may have been caused by physical overload. In contrast, more patients with high HADS scores for anxiety or depression (or both) believed that “their disease was caused by physical overload” and that “flares were triggered by physical effort”, whereas fewer held the opposite belief that “physical activity could reduce flares.” Similarly, more patients with high AHI scores believed that disease flares could be triggered by physical activity, or that their disease may have been caused by environmental factors such as pollution. The belief that “drinking alcohol or smoking caused the disease” was more widely accepted among financially deprived patients (those eligible for an income subsidy from the state).

**Table 3:** Principal associations between patient variables and strongly held beliefs (QuAD score ≥ 7)

	QuAD item	Patient variable	Reference		OR [95% CI]
F4	Physical activity reduces flares	Diagnosis	RA	axSpA	2.15 [1.50–3.08]
O2	Disease caused by smoking	Diagnosis	RA	axSpA	0.60 [0.36–0.96]
D4	Certain foods reduce flares	Gender	Men	Women	2.22 [1.18–4.20]
F1	Disease caused by physical overload	Education	Higher	High school	2.14 [1.30–3.53]
F4	Physical activity reduces flares	Education	Higher	High school	0.42 [0.29–0.60]
O2	Disease caused by smoking	Social deprivation	Not deprived	Deprived	2.04 [1.15–3.62]
D2	Disease caused by alcohol	Social deprivation	Not deprived	Deprived	4.18 [1.19–14.6]
F1	Disease caused by physical overload	Anxiety	HADS-A ≤ 8	HADS-A > 10	2.87 [1.67–4.92]
F2	Flares triggered by physical effort	Anxiety	HADS-A ≤ 8	HADS-A > 10	1.59 [1.03–2.45]
F4	Physical activity reduces flares	Depression	HADS-D ≤ 8	HADS-D > 18	0.58 [0.38–0.88]
F2	Flares triggered by physical effort	Depression	HADS-D ≤ 8	HADS-D > 8	1.49 [1.00–2.23]
F2	Flares triggered by physical effort	Helplessness	AHI < 20	AHI ≥ 20	1.77 [1.23–2.54]
O3	Disease caused by environmental factor	Helplessness	AHI < 20	AHI ≥ 20	2.93 [1.38–6.18]

The codes for the questionnaire items correspond to those listed in Table 2. Data are presented as odds ratios (OR) with 95% confidence intervals (CI)

AHI, Arthritis Helplessness Index; axSpA, axial spondyloarthritis; HADS, Hospital Anxiety and Depression Scale; QuAD, Questionnaire for Arthritis Dialogue; RA, rheumatoid arthritis

Some differences were observed between patients with RA and those with axSpA. Compared to patients with RA, those with axSpA were more likely to believe that taking physical exercise could reduce disease flares, and less likely to believe that their disease was caused by smoking.

These multiple logistic regression analyses were reiterated twice: firstly, by introducing age and gender as forced variables in the models, and then with patient-reported disease activity (visual analogue scale, VAS) and disease duration as forced variables. Although these adjustments altered the odds ratios minimally, the variables retained in the models were not changed (data not shown).

## Discussion

The aim of this study was to develop a questionnaire to evaluate the beliefs of patients with RA and axSpA, and to identify patient characteristics associated with these beliefs. In a large sample of patients with RA or axSpA, our study identified a wide range of patient opinions on their disease and its treatment, and a number of demographic, socioeconomic and psychological factors associated with these opinions.

No individual belief was strongly held by more than half of patients overall. However, more than one-third of patients attributed their disease to psychological or genetic causes, whereas less than 10% attributed it to causes that have little support from medical opinion, such as diet, pollution, smoking, infection, vaccination or alcohol consumption. In contrast, beliefs about lifestyle and CIRDs were often erroneous, perhaps due to inadequate patient education, or because of psychological distress. These beliefs need to be explored by physicians and discussed with the patient to ensure that the patient maintains as healthy a lifestyle as possible.

The diversity of beliefs identified include those that are consistent with current medical opinion, such as the belief that axSpA may have a genetic cause; those that are inconsistent with medical opinion, such as the belief that CIRDs may be caused by vaccination, and those for which there is limited medical consensus or where medical opinion is evolving. In general, the beliefs held were similar between patients with RA and those with axSpA, which may be explained by the similarly unpredictable course of the two diseases, the common core symptoms, and the fact that these patients will usually be cared for in the same healthcare facilities and thus be exposed to the same sources of information. However, patients with axSpA were around twice as likely as those with RA to attribute their disease to a genetic origin, likely reflecting awareness of a strong association between SpA and HLA-B27 [22]. In contrast, when asked to suggest possible causes for their disease, patients with RA were more likely to cite emotional factors. Moreover, patients with axSpA, who were on average younger and had a higher level of education than those with RA, were more likely to believe that physical activity could be beneficial to their disease (in agreement with current medical thinking) and less likely to believe that their disease was caused by smoking.

In this study, we focused on beliefs relating to lifestyle. This choice reflects the fact that these beliefs may be modifiable, potentially leading to changes in lifestyle. Investigation of a patient’s beliefs about lifestyle, and a dialogue about unwarranted beliefs, may help modify behavior and improve health. For example, patients who are convinced of the benefits of physical activity might adopt a regular exercise routine. In addition, dispelling unwarranted fears about the risks of vaccination may encourage patients to be vaccinated against infectious diseases.

In some cases, different groups of patients held opposing beliefs, such as those relating to the impact of physical activity or diet on disease flares. For example, the proportion of patients who believed that physical activity triggered their disease flares (35.5%) was comparable to the proportion of those who held the opposite opinion, that physical activity reduced their flares (36.5%). It should be noted, however, that the two items are not wholly comparable, since they are phrased somewhat differently: the deleterious belief referring to “physical effort” (passive) and the beneficial belief referring to “doing sport or physical activity” (active). Nevertheless, the characteristics of these two groups of patients were different: those who considered physical activity to be detrimental were more frequently anxious or depressed and expressed a high helplessness score, possibly indicating that this belief was associated with psychological distress. In contrast, patients who believed that physical activity was beneficial tended to be better educated, less depressed, and to rate their disease activity as low. With respect to this particular belief, it is noteworthy that medical opinion has evolved over recent years—whereas in the twentieth century many physicians had a conservative approach to exercise in patients with CIRDs, a consensus has now emerged that exercise and sports activities are helpful in the short- and long-term management of disease, which is reflected in current practice guidelines [23, 24].

The study has a number of limitations that should be considered when interpreting the results. Firstly, participation of rheumatologists was voluntary and not remunerated, and physicians with a hospital practice were over-represented. This may have influenced the representativeness of the patient sample included. However, the age and gender distributions of enrolled patients were similar to those of the overall RA [25] or axSpA [26] populations. Nevertheless, the proportion of patients in this study who were undergoing treatment with biological DMARDs was much higher than national figures (75%, versus 14% of all French RA patients [25] and 26% of all axSpA patients [26]). Another factor to be considered in interpretation of these data relates to the choice of the cut-off value for the identification of “strongly held” beliefs (≥ 7 on the VAS). This choice was purely empirical and, in the absence of any known disease characteristic with which these beliefs are correlated, we do not believe that it is realistic to attempt any psychometric calibration of the VAS. Use of a threshold lower than 7 would clearly generate higher percentages of “believers”. Finally, it is important to note that the items of the QuAD were derived from a qualitative survey of French patients [11]. It is possible that patients in other countries or cultures would have different concerns [27, 28], which would be interesting to evaluate in future studies.

We believe that exploring patient beliefs about disease and treatment with a questionnaire such as the QuAD is useful for the physician in several ways; for example, to facilitate dialogue with the patient and to help patients understand their disease and form realistic treatment expectations. In particular, we believe that physicians should discuss lifestyle beliefs with their patients in order to dissipate unwarranted concerns and unfounded beliefs, and to encourage the adoption of healthy lifestyles. Facilitating physician-patient dialogue in this way would be expected to improve the overall quality of care, and to encourage the patient to become an active partner in setting and achieving treatment goals. In addition, the questionnaire may be a useful component of therapeutic education programs for structuring debate about disease perceptions. Finally, patients may feel that their perceptions and concerns about their disease are not considered important or discussed by their physician, and the availability of the QuAD might help to address this need. At a population level, the questionnaire could also describe shifts in beliefs over time (following awareness campaigns, for example).

In conclusion, it is important to understand and discuss patients’ beliefs about inflammatory rheumatic diseases in order to optimize the quality of care. The QuAD provides a simple tool to help achieve this, and merits further assessment.

## Electronic supplementary material

ESM 1 (DOCX 72 kb)
