# Correction to: Development and application of a questionnaire to assess patient beliefs in rheumatoid arthritis and axial spondyloarthritis

**DOI:** 10.1007/s10067-018-4241-9

**Published:** 2018-08-24

**Authors:** Laure Gossec, Francis Berenbaum, Pierre Chauvin, Christophe Hudry, Gabrielle Cukierman, Thibault de Chalus, Caroline Dreuillet, Vincent Saulot, Sabine Tong, Françoise Russo-Marie, Jean-Michel Joubert, Alain Saraux

**Affiliations:** 1 Sorbonne Université, Institut Pierre Louis d’Epidémiologie et de Santé Publique (INSERM UMRS 1136), GRC-UPMC 08 (EEMOIS), Paris, France; 2 Service de rhumatologie, Hôpital Pitié Salpêtrière, 47–83 Boulevard de l’Hôpital, 75013 Paris, France; 3 Sorbonne Université, INSERM, Paris, France; 4 Department of Rheumatology, Hôpital Saint-Antoine, AP-HP, Paris, France; 5 Sorbonne Université, INSERM, Institut Pierre Louis d’Epidémiologie et de Santé Publique (UMRS 1136), Department of Social Epidemiology, Sorbonne Universités, Paris, France; 6 Department of Rheumatology, Hôpital Cochin, AP-HP, Paris, France; 7 UCB Pharma, Colombes, France; 8 Arthritis Fondation Courtin, Neuilly-Sur-Seine, France; 9 AXONAL-BIOSTATEM, Nanterre, France; 10 Department of Rheumatology, CHU de la Cavale-Blanche, Brest, France; 11 INSERM, LabEx IGO, UMR1227, Lymphocytes B et Autoimmunité, Université de Brest, Brest, France

**Correction to: Clinical Rheumatology**

10.1007/s10067-018-4172-5

The article “Development and application of a questionnaire to assess patient beliefs in rheumatoid arthritis and axial spondyloarthritis”, written by Laure Gossec, Francis Berenbaum, Pierre Chauvin, Christophe Hudry, Gabrielle Cukierman, Thibault de Chalus, Caroline Dreuillet, Vincent Saulot, Sabine Tong, Françoise Russo-Marie, Jean-Michel Joubert and Alain Saraux, was originally published electronically on the publisher’s internet portal (currently SpringerLink) on 12 June 2018 without open access.

With the author(s)’ decision to opt for Open Choice the copyright of the article changed on August 2018 to © The Author(s) 2018 and the article is forthwith distributed under the terms of the Creative Commons Attribution 4.0 International License (http://creativecommons.org/licenses/by/4.0/), which permits use, duplication, adaptation, distribution and reproduction in any medium or format, as long as you give appropriate credit to the original author(s) and the source, provide a link to the Creative Commons license and indicate if changes were made.

The original article was corrected.

