# Supplementary material for: Development and application of a questionnaire to assess patient beliefs in rheumatoid arthritis and axial spondyloarthritis
Source: Clin Rheumatol. 2018 Jun 12;37(10):2649–57. doi: 10.1007/s10067-018-4172-5 (PMC6154088; doi:10.1007/s10067-018-4172-5)
Supplement: Supplementary file 1 — (DOCX 72 kb) [file 10067_2018_4172_MOESM1_ESM.docx]

**Development and Application of a Questionnaire to Assess Patient Beliefs in Rheumatoid Arthritis and Axial Spondyloarthritis**

**Journal:** Clinical Rheumatology

**Authors:** Laure Gossec, Francis Berenbaum, Pierre Chauvin, Christophe Hudry, Gabrielle Cukierman, Thibault de Chalus, Caroline Dreuillet, Vincent Saulot, Sabine Tong, Françoise Russo-Marie, Jean-Michel Joubert, Alain Saraux

**Correspondence to:** Laure Gossec, Service de rhumatologie, Hôpital Pitié Salpêtrière, 47–83 Boulevard de l'Hôpital, 75013, Paris, France. Tel: +33 1 42 17 84 21; Fax: +33 42177959; Email: [laure.gossec@aphp.fr](mailto:laure.gossec@aphp.fr)

SUPPLEMENTARY DATA

**Online Resource 1** Factors associated with beliefs about physical activity

| **F1: I think that my disease was triggered by physical overload.** | | | | |
| --- | --- | --- | --- | --- |
|  | | **N (%)** | **Univariate OR**  **[95% CI]** | **Multivariate OR** |
| **Gender** | *p*=0.035 |  | NS |  |
| Men | 30/208 (14.4%) | 1 |  |  |
| Women | 79/365 (21.6%) | 1.64 [1.04–2.59] |  |  |
| **Educational level** | | *p* <0.001 |  |  |
| Primary | | 25/223 (11.2%) | 1 | 1 |
| Secondary | | 12/84 (14.3%) | 1.26 [0.60–2.63] | 1.09 [0.50–2.38] |
| Tertiary | | 85/350 (24.3%) | 2.53 [1.56–4.09] | 2.14 [1.30–3.53] |
| **Occupation** | | *p*=0.044 |  | NS |
| Professional | | 13/115 (11.3%) | 1 |  |
| Tradesmen/craftsmen | | 17/110 (15.5%) | 1.42 [0.65–3.08] |  |
| Blue/white collar | | 64/298 (21.5%) | 2.14 [1.13–4.05] |  |
| **Living status** | | *p* <0.001 |  |  |
| Living with others | | 82/547 (15.0%) | 1 | 1 |
| Living alone | | 39/108 (36.1%) | 3.27 [2.07–5.16] | 2.44 [1.49–3.98] |
| **Municipality of residence** | | *p*=0.042 |  | NS |
| Metropolitan Paris | | 14/130 (10.8%) | 0.47 [0.26–0.86] |  |
| Other urban | | 95/468 (20.3%) | 1 |  |
| Rural | | 15/65 (23.1%) | 1.07 [0.57–2.02] |  |
| **Disease duration** | | *p*=0.002 |  |  |
| >10 years | | 37/282 (13.1%) | 1 | 1 |
| 5–10 years | | 25/142 (17.6%) | 1.43 [0.82–2.49] | 1.23 [0.69–2.21] |
| ≤5 years | | 36/129 (27.9%) | 2.56 [1.53–4.29] | 2.08 [1.24–3.48] |
| **HADS Anxiety Score** | | *p* <0.001 |  |  |
| <8 (not anxious) | | 26/289 (9.0%) |  | 1 |
| 8–10 (possible anxiety) | | 40/166 (24.1%) | 3.26 [1.91–5.58] | 2.71 [1.56–4.72] |
| >10 (clinical anxiety) | | 56/198 (28.3%) | 3.94 [2.37–6.54] | 2.87 [1.67–4.92] |
| **HADS Depression Score** | | *p*=0.006 |  | NS |
| <8 (not depressed) | | 79/489 (16.2%) | 1 |  |
| >8 (possible/clinical depression) | | 44/166 (26.5%) | 1.80 [1.18–2.73] |  |
| **AHI Score** | | *p* <0.001 |  | NS |
| <20 | | 60/419 (14.3%) | 1 |  |
| ≥20 | | 55/211 (26.1%) | 2.08 [1.38–3.14] |  |
| **Perceived disease activity (patient)** | | *p*=0.005 |  | NS |
| ≤30 | | 40/304 (13.2%) | 1 |  |
| 30–60 | | 34/172 (19.8%) | 1.62 [0.98–2.67] |  |
| >60 | | 31/116 (26.7%) | 2.37 [1.40–4.02] |  |
| **Difference patient/physician PGA** | | *p*=0.011 |  | NS |
| <-2 | | 10/67 (14.9%) | 1 |  |
| -2–2 | | 56/377 (14.9%) | 0.99 [0.48–2.05] |  |
| >2 | | 35/132 (26.5%) | 2.02 [0.93–4.37] |  |
| **F2: I think that flare-ups of my disease are triggered by physical effort** | | | | |
|  | | **N (%)** | **Univariate OR** | **Multivariate OR** |
| **Diagnosis** | | *p*=0.002 |  | NS |
| RA | | 76/425 (17.9%) | 1 |  |
| axSpA | | 47/238 (19.7%) | 1.69 [1.22–2.34] |  |
| **Age** | | *p* <0.001 |  |  |
| ≥60 years | | 51/221 (23.1%) | 1 | 1 |
| <40 years | | 43/93 (46.2%) | 2.95 [1.77–4.93] | 2.81 [1.66–4.75] |
| 40–50 years | | 50/118 (42.4%) | 2.49 [1.54–4.02] | 2.10 [1.25–3.51] |
| 50–60 years | | 59/137 (43.1%) | 2.60 [1.64–4.11] | 2.22 [1.39–3.53] |
| **Occupation** | | *p*=0.007 |  | NS |
| Professional | | 34/115 (29.6%) | 1 |  |
| Tradesmen/craftsmen | | 33/110 (30.0%) | 1.01 [0.57–1.78] |  |
| Blue/white collar | | 129/297 (43.4%) | 1.81 [1.14–2.87] |  |
| **Eligibility for low income benefit** | | *p*=0.019 |  | NS |
| Ineligible | | 193/563 (34.3%) | 1 |  |
| Eligible | | 38/79 (48.1%) | 1.76 [1.10–2.82] |  |
| **Living status** | | *p*=0.009 |  | NS |
| Alone | | 50/108 (47.2%) | 1 |  |
| With another | | 184/549 (33.5%) | 0.57 [0.38–0.87] |  |
| **Nationality** | | *p*=0.045 |  |  |
| French born | | 205/600 (34.2%) | 1 | 1 |
| Naturalized French | | 19/37 (51.4%) | 2.07 [1.06–4.03] | 2.20 [1.07–4.55] |
| Other | | 11/23 (47.8%) | 1.80 [0.78–4.14] | 2.78 [1.12–6.90] |
| **Municipality of residence** | | *p*=0.011 |  |  |
| Metropolitan Paris | | 36/131 (27.5%) | 1 | 1 |
| Other urban | | 176/469 (37.5%) | 1.58 [1.03–2.41] | 2.03 [1.27–3.25] |
| Rural | | 24/65 (36.9%) | 1.52 [0.81–2.86] | 1.91 [0.95–3.81] |
| **HADS Anxiety Score** | | *p* <0.001 |  |  |
| <8 (not anxious) | | 78/289 (27.0%) | 1 | 1 |
| 8–10 (possible anxiety) | | 64/166 (38.6%) | 1.73 [1.15–2.60] | 1.54 [1.00–2.36] |
| >10 (clinical anxiety) | | 90/200 (45.0%) | 2.22 [1.52–3.24] | 1.59 [1.03–2.45] |
| **HADS Depression Score** | | *p* <0.001 |  |  |
| <8 (not depressed) | | 155/489 (31.7%) | 1 | 1 |
| >8 (possible/clinical depression) | | 79/168 (47.0%) | 1.85 [1.30–2.63] | 1.49 [1.00–2.23] |
| **AHI Score** | | *p* <0.001 |  |  |
| <20 | | 121/419 (28.9%) | 1 | 1 |
| ≥20 | | 101/212 (47.6%) | 2.19 [1.56–3.08] | 1.77 [1.23–2.54] |
| **Perceived disease activity (patient)** | | *p*=0.002 |  | NS |
| ≤30 | | 91/306 (29.7%) | 1 |  |
| 30–60 | | 66/172 (38.3%) | 1.44 [0.98–2.14] |  |
| >60 | | 56/115 (48.7%) | 2.13 [1.38–3.30] |  |
| **Difference patient/physician PGA** | | *p*=0.023 |  | NS |
| <-2 | | 22/67 (32.8%) | 1 |  |
| -2–2 | | 123/377 (32.6%) | 0.98 [0.57–1.71] |  |
| >2 | | 61/132 (46.2%) | 1.710 [0.93–3.16] |  |
| **F4: I think that doing sport or a physical activity reduces my flare-ups.** | | | | |
|  | | **N (%)** | **Univariate OR** | **Multivariate OR** |
| **Diagnosis** | | *p* <0.001 |  |  |
| RA | | 135/430 (31.4%) | 1 | 1 |
| axSpA | | 109/238 (45.8%) | 1.83 [1.32–2.54] | 2.15 [1.50–3.08] |
| **Educational level** | | *p* <0.001 |  |  |
| Primary | | 114/223 (51.1%) | 1 | 1 |
| Secondary | | 29/88 (33.0%) | 0.78 [0.47–1.29] | 0.60 [0.35–1.02] |
| Tertiary | | 98/351 (27.9%) | 2.11 [1.26–3.53] | 1.45 [0.84–2.50] |
| **Living status** | | *p*=0.032 |  | NS |
| Alone | | 37/103 (35.9%) | 1 |  |
| With another adult but no children | | 82/250 (32.8%) | 0.88 [0.54–1.42] |  |
| With another adult and children | | 41/81 (50.6%) | 1.86 [1.03–3.36] |  |
| With children but no other adult | | 61/153 (39.9%) | 1.20 [0.72–2.01] |  |
| **HADS Depression Score** | | *p*=0.002 |  |  |
| <8 (not depressed) | | 198/490 (40.4%) | 1 | 1 |
| >8 (possible/clinical depression) | | 46/170 (27.1%) | 0.54 [0.37–0.80] | 0.58 [0.38–0.88] |

Odds ratios [95% CIs] are derived from multiple logistic regression analyses. Univariate analyses were performed using a χ² test. All p values were derived from univariate analyses. The belief that bad posture or immobility triggered disease flares (F3) was not associated with any particular patient characteristics. AHI: Arthritis Helplessness Index; HADS: Hospital Anxiety and Depression Scale; NS: not significant; OR: odds ratio; PGA: physician’s global assessment.

**Online Resource 2** Factors associated with beliefs about food or diet

| **D2: I think that drinking alcohol (even moderately) triggered my disease** | | | | | | | |
| --- | --- | --- | --- | --- | --- | --- | --- |
|  | | **N (%)** | | **Univariate OR** | | **Multivariate OR** | |
| **Eligibility for low income benefit** | | *p* = 0.024 | |  | |  | |
| Ineligible | | 7/560 (1.3%) | | 1 | | 1 | |
| Eligible | | 4/77 (5.2%) | | 4.22 [1.21–14.75] | | 4.18 [1.19–14.60] | |
| **D3: I think that eating certain foods triggers my flare-ups** | | | | | | | |
|  | | **N (%)** | | **Univariate OR** | | **Multivariate OR** | |
| **HADS Anxiety score** | | *p* = 0.029 | |  | |  | |
| >10 (clinical anxiety) | | 12/200 (6.0%) | | 1 | | 1 | |
| 8–10 (possible anxiety) | | 24/164 (14.6%) | | 2.68 [1.29–5.53] | | 2.59 [1.26–5.36] | |
| <8 (not anxious) | | 31/290 (10.7%) | | 1.87 [0.94–3.74] | | 1.88 [0.94–3.75] | |
| **HADS Depression Score** | | *p* = 0.065 | |  | | NS | |
| >8 (possible/clinical depression) | | 11/168 (6.5%) | | 1 | |  | |
| <8 (not depressed) | | 56/488 (11.5%) | | 1.88 [0.96–3.68] | |  | |
| **D4: I think that eating certain foods reduces my flare-ups** | | | | | | | |
|  | | **N (%)** | | **Univariate OR** | | **Multivariate OR** | |
| **Gender** | | *p* = 0.009 | |  | |  | |
| Men | | 12/209 (5.7%) | | 1 | | 1 | |
| Women | | 47/366 (12.8%) | | 2.41 [1.25–4.66] | | 2.22 [1.18–4.20] | |
| **Treatment** | | *p* = 0.003 | |  | |  | |
| Biological DMARDs | | 33/419 (7.8%) | | 1 | | 1 | |
| Others | | 23/138 (16.7%) | | 2.36 [1.33–4.17] | | 0.44 [0.25–0.77] | |

Odds ratios [95% CIs] are derived from multiple logistic regression analyses. Univariate analyses were performed using a χ² test. The belief that the disease was caused by dietary factors (D1) was not associated with any particular patient characteristics. DMARD: disease-modifying antirheumatic drug; HADS: Hospital Anxiety and Depression Scale; NS: not significant; OR: odds ratio.

**Online Resource 3** Factors associated with beliefs about other lifestyle factors

| **O2: I think that smoking (even moderately) triggered my disease** | | | | |  |  |  |
| --- | --- | --- | --- | --- | --- | --- | --- |
|  | **N (%)** | **Univariate OR** | **Multivariate OR** | | | |  |
| **Diagnosis** | *p* = 0.038 |  |  | | | |  |
| Axial spondyloarthritis | 4/237 1.7%) | 1 | 1 | | | |  |
| Rheumatoid arthritis | 26/419 (6.2%) | 1.62 [1.03–2.54] | 1.68 [1.04–2.78] | | | |  |
| **Disease duration** | *p* = 0.012 |  |  | | | |  |
| >10 years | 13/282 (4.6%) | 1 | 1 | | | |  |
| 5–10 years | 6/140 (4.3%) | 1.60 [0.92–2.78] | 1.50 [0.89–2.52] | | | |  |
| ≤5 years | 8/128 (6.3%) | 2.23 [1.31–3.82] | 1.96 [1.09–3.50] | | | |  |
| **Eligibility for low income benefit** | *p* = 0.003 |  |  | | | |  |
| Ineligible | 23/557 (4.1%) | 1 | 1 | | | |  |
| Eligible | 6/76 (7.9%) | 2.28 [1.32–3.93] | 2.04 [1.15–3.62] | | | |  |
| **Living status** | *p* <0.001 |  |  | | | |  |
| Living with others | 22/542 (4.1%) | 1 | 1 | | | |  |
| Living alone | 8/106 (7.5%) | 2.95 [1.84–4.73] | 2.82 [1.72–4.62] | | | |  |
| **Marital status** | *p* = 0.021 |  | NS | | | |  |
| Single | 8/146 (5.5%) | 1 |  | | | |  |
| Married | 22/504 (4.4%) | 0.59 [0.37–0.92] |  | | | |  |
| **O3: I think that my disease was triggered by something in the environment, like pollution** | | | | | |  |  |
|  | **N (%)** | **Univariate OR** | **Multivariate OR** | | | |  |
| **Occupation** | *p* = 0.05 |  | | NS | | | |
| Blue/white collar | 22/298 (7.4%) | 1 | |  | | | |
| Tradesmen/craftsmen | 4/109 (3.7%) | 0.47 [0.16–1.398] | |  | | | |
| Professional | 1/115 (0.9%) | 0.11 [0.02–0.83] | |  | | | |
| **Living status** | 0.004 |  |  | | | |  |
| Living with others | 26/544 (4.8%) | 1 | 1 | | | |  |
| Living alone | 13/107 (12.1%) | 2.79 [1.38–5.62] | 2.49 [1.19–5.25] | | | |  |
| **AHI score** | <0.001 |  |  | | | |  |
| <20 | 14/418 (3.3%) | 1 | 1 | | | |  |
| ≥20 | 23/208 (11.1%) | 3.50 [1.76–6.95] | 2.93 [1.38–6.18] | | | |  |
| **Difference patient/physician PGA** | 0.010 |  |  | | | |  |
| >2 | 15/132 (11.4%) | 1 | 1 | | | |  |
| -2–2 | 16/376 (4.3%) | 0.35 [0.17–0.73] | 0.35 [0.13–0.92] | | | |  |
| <-2 | 2/66 (3.0%) | 0.24 [0.05–1.10] | 0.18 [0.03–1.05] | | | |  |

Odds ratios [95% CIs] are derived from multiple logistic regression analyses. Univariate analyses were performed using a χ² test. The belief that fatigue triggered disease flares (O1) was not associated with any particular patient characteristics. AHI: Arthritis Helplessness Index; NS: not significant; OR: odds ratio; PGA: physician’s global assessment.
